# How Do I Narrate My Marriage: The Relationship Between Attachment Orientation and Quality of Autobiographical Memory

**DOI:** 10.3389/fpsyg.2018.02107

**Published:** 2018-11-01

**Authors:** Yan Wang, Qianrong Wang, Dahua Wang, Brooke C. Feeney

**Affiliations:** ^1^Faculty of Psychology, Beijing Normal University, Beijing, China; ^2^Faculty of Social Sciences, The University of Hong Kong, Hong Kong, China; ^3^Institute of Developmental Psychology, Beijing Normal University, Beijing, China; ^4^Department of Psychology, Carnegie Mellon University, Pittsburgh, PA, United States

**Keywords:** attachment, autobiographical memory, coherence, vividness, narrative

## Abstract

Attachment orientations play important roles in the generation of emotional autobiographical memory (AM). However, little research has considered the quality of autographical narratives, which may reflect the structure and content of internal working models (IWMs) of attachment. The purpose of the current study was to investigate the relationship between attachment orientations and narrative quality of marriage-related autobiographical memories. Ninety-four married adults were asked to retrieve two episodes of emotional autobiographical memories. The coherence and vividness of their narratives were then coded. Results indicated that adults who were highly avoidant were more likely to present their memories in a less coherent way and to describe negative memories with more perceptual details. In contrast, attachment anxiety was associated with lower vividness of negative memories. The current findings suggest that an attachment schematic-processing strategy was used in narrating the attachment-related experiences.

## Introduction

Autobiographical memory (AM) represents the personal memory of ones’ experiences and reflects the history of an individual’s life (Conway, 1992). Given its self-referring nature (Brewer, 1986), the construction of AM should be influenced by the self (Conway et al., 2004). This investigation considers attachment orientation, which reflects internal working models (IWMs) of the self and others, as important predictors of the quality of autobiographical narratives.

Although some efforts have been made to explore how attachment shapes one’s AM in adulthood, there are still unclear issues. First, previous studies usually examine the phenomenological experiences associated with remembering, such as vividness and coherence (e.g., Sutin and Gillath, 2009). However, these conscious evaluations after self-regulation do not reveal attachment influences on the actual content of AM. Additionally, most existent studies focus on childhood memories (e.g., Mikulincer and Orbach, 1995). Little is known about whether attachment orientation is related to the way individuals recall their memories about marriage, which is a prototypical attachment relationship in adulthood. This study contributes to filling an important gap in the literature by investigating the relationship between attachment orientations (which reflect IWMs of attachment) and the narrative quality of relationship-related AM among long-married adults.

### Attachment and Autobiographical Memory

Attachment researchers have described the function of IWMs of attachment related to information processing (e.g., Bowlby, 1969; Collins et al., 2004; Bretherton and Munholland, 2008; Dykas and Cassidy, 2011). Specifically, IWMs (e.g., representations of the self and interactions with close others during attempts to attain felt security, as well as the typical outcomes of those attempts) are regarded as schemas that guide an individual’s perception, interpretation, memory, and reaction to the situations they encounter, especially ones that involve stress, threat, or vulnerability. Attachment research suggests that individual differences in IWMs (also referred to one’s attachment orientation) are best described through two dimensions, namely, attachment avoidance and attachment anxiety (Brennan et al., 1998; Fraley and Spieker, 2003). The attachment anxiety dimension assesses fear of abandonment and distress about separation, while the attachment avoidance dimension assesses discomfort with intimacy or dependence on others (Mikulincer and Shaver, 2016).

Relevant to AM, individuals with different attachment orientations are likely to adopt different coping strategies when encountering attachment-related stimuli or experiences (Shaver and Mikulincer, 2002; Mikulincer and Shaver, 2016). Specifically, avoidant adults tend to deactivate their attachment system by suppression of attachment-related thoughts and emotions (e.g., Edelstein et al., 2012; Ben-Naim et al., 2013; Andriopoulos and Kafetsios, 2015), whereas anxious adults tend to hyperactivate their attachment system by showing intensified expression of distress and attachment-related worries (Gillath et al., 2005; Rognoni et al., 2008).

A few studies focused on the self-reported phenomenological experiences associated with remembering provide some support for the influence of these regulatory strategies on AM. For example, highly avoidant individuals who attempt to down-regulate emotions report attachment-related autobiographical memories that have less emotional intensity (Mikulincer and Orbach, 1995; Haggerty et al., 2010) and are less coherent than less avoidant individuals (Sutin and Gillath, 2009), representing their deactivation of the attachment system. In contrast, anxious adults are more likely to be highly aroused by negative attachment-related autobiographical memories and to evaluate these memories as vivid and pictorial (Öner and Gülgöz, 2016).

Although these studies have provided a preliminary investigation of the role that attachment plays in the construction of AM (e.g., Sutin and Gillath, 2009; Öner and Gülgöz, 2016), the self-report methodology used in these studies is likely to prompt participants to retrieve memories through a particular filter (Panattoni and McLean, 2017). In addition, it may capture a participant’s conscious evaluation of features of a narrated experience and may be influenced by self-regulation (Alea et al., 2004; Panattoni and McLean, 2017). Instead, actual narratives may provide a useful tool for revealing the individuals’ spontaneous reaction in the retrospection (Alea et al., 2004) and may show greater potential to reveal unconscious features of autobiographical memories such as how the information is processed (Waters et al., 2014; Adler et al., 2016; Grysman et al., 2017; Panattoni and McLean, 2017).

### Attachment and the Quality of Autobiographical Narratives

Given that Bowlby argued that narratives of past attachment experiences are critical to the development and expression of a coherent IWM (Bowlby, 1969), it is valuable to examine the relationship between attachment orientations and narrative quality of AM. This will enhance our understanding of attachment effects on the construction of one’s experiences. Autobiographical narratives are likely to reflect the structure and content of IWMs of attachment (Kelly, 2015) and may therefore reveal the attachment-schema-driven processing of attachment-related experiences without conscious, self-regulation. When recalling past attachment-related experiences, insecure individuals rely on their negative IWMs of others as being unavailable and unresponsive (avoidant) or of the self as being unworthy of love and affection from others (anxious). Therefore, they tend to show less efficient emotion regulation (Mikulincer and Orbach, 1995) and process relationship-related information in a more negative fashion than secure adults who are low in both anxiety and avoidance (Feeney and Cassidy, 2003; Dykas and Cassidy, 2011).

Recently, several studies focused on children’s or adolescents’ memory in relationship with their parents have examined attachment effects on the construction of autobiographical narratives (e.g., Kelly, 2015). Consistent with the schema-driven processing function of IWMs (Dykas and Cassidy, 2011), these studies showed that both anxious-insecure and avoidant-insecure individuals, who hold negative attachment schema, narrate their autobiographical memories about events that happened with their parents with poorer structure (e.g., shorter and less coherent narratives) than their secure counterparts (Zaman and Fivush, 2013; Kelly, 2015).

However, little is known about the function of attachment in the construction of adults’ autobiographical narratives about events that happened with a spouse, who becomes individual’s primary attachment figure in adulthood (Ross and Spinner, 2001). It is important to examine whether adults process their autobiographical narratives about romantic relationships schematically, in ways consistent with their attachment representations, as Bowlby postulated that attachment processes characterize individuals “from the cradle to the grave” (Bowlby, 1973, p. 208).

Furthermore, the existent studies with children or adolescents assess only narrative structure (e.g., coherence), yet additional aspects of autobiographical narratives, such as the extent to which the narratives are detailed (e.g., vividness), would likely provide a more complete picture of the role of attachment orientations in the construction of AM. In the present study, two widely used and valid indicators of retrieval quality – vividness and coherence – were used to assess the quality of autobiographical narratives of the marriages of older adults (Nelson et al., 2008). These are feature of high-quality narratives (Reese et al., 2011) and reflect a good memory of specific information (Greenberg and Rubin, 2003; Fivush, 2008). Considering both of these indicators, we can examine whether attachment orientation shapes the level of details provided in AMs of marriage, as well as the extent to which the information is organized in a coherent way.

### Current Study

Given that the function of attachment in narrating autobiographical memories of relationships with romantic partners have not been explored (nor have autobiographical memories of any type of relationship in adulthood), this study aimed to investigate the relationship between attachment orientations and the quality of autobiographical narratives of older adults in long-term marriages. A sample of long-married adults, who were in a relatively stable marital relationship for at least 20 years and had a large repertoire of lifetime memories, were recruited in the current study. They were asked to retrieve both a positive and a negative AM of an experience with their spouses. Considering that verbal ability and rehearsal frequency of the memory could affect the narrative discourse (e.g., Fitzgerald and Broadbridge, 2013), both were assessed as control variables. Each narrative was coded for the quality of the AM on both vividness and coherence.

Since autobiographical narratives are presumed to reflect the structure and content of IWMs of attachment (Kelly, 2015), we expected that adults would narrate their attachment-related memories schematically. Specifically, avoidant adults possess negative attachment schemas involving discomfort with intimacy and an inability to depend on others. They are likely to deactivate their attachment system at the time relationship-related memories are being formed (particularly distressing ones) and later when narrating the experience (Bretherton and Munholland, 2008; Mikulincer and Shaver, 2016). Thus, we predicted that greater attachment avoidance would be associated with less coherent and less vivid autobiographical narratives of both positive and negative relationship experiences with one’s spouse. In contrast, anxious adults possess schemas involving concerns about rejection and feelings of unworthiness, which lead them to hyperactivate the attachment system, intensifying their experiences of distress and sensitizing them to signals of potential threats (Shaver and Mikulincer, 2002). These individuals may become especially distressed when processing negative information (Mikulincer and Shaver, 2016). Thus, we hypothesized that anxious attachment would predict less fluent (less coherent) and less detailed (less vivid) autobiographical narratives of negative relationship events.

## Materials and Methods

### Participants

Participants were part of a larger research project on marital attachment among older adults. A total of 697 married older adults (including 263 couples and 171 individuals) who had been in their current marriage for at least 20 years were recruited from communities in Beijing, China. From this sample, only individuals were invited by phone to participate in the current study. Seventy of those contacted (56 females; mean age = 68.14 ± 5.16 years) either did not answer the phone or declined. A total of 101 adults (62 females; mean age = 65.50 ± 3.98 years) were contacted successfully and agreed to participate. They were younger than the individuals who did not join the study [*t*(169) = -3.96, *p* < 0.001], but there were no significant differences in duration of marriage [*t*(169) = -1.81, *p* = 0.073] and years of education [*t*(169) = 1.79, *p* = 0.076]. The Clock–Drawing Test (CDT; Shulman et al., 1986) was used to exclude participants with cognitive impairment. Seven adults who scored less than three on the CDT were excluded from the study. The final sample included 94 participants (37 males and 57 females) with ages ranging from 58 to 74 years (mean age = 65.33 ± 3.91 years). Participants had an average of 12.73 years of education (*SD* = 2.70) and their average duration of marriage was 37.68 years (*SD* = 4.73).

A power analysis was conducted using the effect size estimates of previous studies examining the relationship between attachment and autobiographical narratives in childhood (Kelly, 2015). The analysis with G^∗^Power 3.1 (Faul et al., 2007) indicated a minimum sample size of 74 to detect the predictive effect of attachment orientations at *p* < 0.05 with power = 0.80, assuming a medium effect size of 0.11.

### Procedure

Participants were invited to the laboratory and informed that they were participating in a study about memories of marital life. After obtaining informed consent, they were interviewed individually and audiotaped by a trained experimenter.

Participants initially provided demographic information and completed assessments of their verbal ability using the vocabulary subtest from the Wechsler’s Adult Intelligence Scale-Revised for China (WAIS-RC; Gong, 1983) and of their marital attachment using the Older Adults’ Marital Attachment Scale. They were then permitted 5 min to complete a picture description task (Rudoy et al., 2009), which was designed as a practice task to induce a detail-based retrieval orientation. In this task, participants were shown a photograph in which two people were standing in front of a store, and they were asked to provide a detailed description of the photograph. If a participant failed to describe details about the photograph (e.g., “A couple is buying something in a store”), the experimenter prompted him/her with examples (e.g., “On a sunny morning, an old silver-haired couple is standing in front of a small store selling toys, drinks and snacks”), followed by a request for them to describe it again.

Finally, participants completed two 5 min deep-recall tasks of autobiographical memories of experiences with the spouse, one consisting of a detailed recall of a positive marital event and one of a negative marital event. Participants’ responses were audio recorded as they provided the narratives. After each AM interview, the participants’ rehearsal frequency of memory was assessed with a single question, “Since it happened, I have thought or talked about this event” (from the Autobiographical Memory Questionnaire, Talarico et al., 2004) on a 7-point scale (1 represents “not at all” and 7 represents “more than for any other memory”). The order in which participants recalled the positive and negative events was counterbalanced. A backwards digit recall test (in which participants were asked to recall, in reverse order, sequences of digits spoken aloud by the experimenter) was inserted as a filler task between the two interviews of AM in order to diminish the potential influence of the former interview on the latter one.

### Measures

#### Marital Attachment

Marital attachment anxiety and avoidance were assessed using the two subscales of the Older Adults’ Marital Attachment Scale (OAMAS; Zhai et al., 2010). Six items assessed marital attachment anxiety (e.g., “I worry that my wife/husband won’t care about me as much as I care about her/him.”), and six items assessed marital attachment avoidance (e.g., “I don’t like to stay too close to my wife/husband.”). Items were rated on a Likert scale ranging from 1 (“strongly disagree”) to 7 (“strongly agree”). Previous studies have validated this measure in Chinese elderly (Wang et al., 2012, 2014). In this study, Cronbach’s alphas for the subscales of anxiety and avoidance were 0.67 and 0.83, respectively.

#### Narrative Coding

Both positive and negative AM interviews of each participant were transcribed verbatim. A narrative coding system was developed to capture the core properties of narrative vividness and coherence. The coders were graduate students who were blind to the participants’ demographic information, attachment orientations, and aims of the study.

Narratives were initially divided into sentences. Sentences of greeting and ending that were not relevant to the memory task were treated as invalid. Two coders independently identified valid sentences, and instances of inconsistent coding were discussed until both reached an agreement. The total number of valid sentences was calculated for each participant. The average numbers of valid sentences for positive and negative memories was 66.47 (*SD* = 35.35) and 71.92 (*SD* = 45.09), respectively.

##### Vividness

According to the definition of vividness (Greenberg and Rubin, 2003), the extent to which the narrative provided perceptual details was assessed. A word or phrase in a valid sentence was identified as a vivid segment if it was: (1) a concrete description of the context of the event (e.g., weather, date, place, and characters), (2) a detailed description of verbal communication that occurred during the event (e.g., dialog and monolog), or (3) a specific description of subjective feelings regarding the event (e.g., emotions felt or expressed). The sum of vivid segments was calculated for use in data analysis. A subsample of 50% of randomly selected transcripts were coded by both raters independently, and the ICC was 0.96 for the positive memory and 0.96 for the negative memory. The remaining 50% of the transcripts were divided equally between the two raters and coded independently. The average numbers of vivid segments was 38.93 (*SD* = 17.92) for the positive memory and 34.79 (*SD* = 18.07) for the negative memory.

##### Coherence

According to the definition of coherence used in the study of the phenomenology of AM (Talarico et al., 2004), the extent to which the narrative was told in a logical and fluent way was assessed. Sentences were coded as coherent if they did not contain inconsistent content with regard to the whole story (e.g., distracting information or digressions that made the theme difficult to understand), incoherent plot as related to the whole story (e.g., incorrect chronological orders or illogical sequences), and disfluent speech (e.g., an unusual pause that disrupts the flow of the narrative or nonsense words). The total number of coherent sentences was calculated for each memory transcript. A subsample of 50% of randomly selected transcripts were coded by both raters independently, and the ICC was 0.97 for the positive memory and 0.93 for the negative memory. The remaining 50% of the transcripts were divided equally between the two raters. The average of coherent sentences in positive and negative memories were 54.05 (*SD* = 30.61) and 58.84 (*SD* = 37.73), respectively.

## Results

### Preliminary Analyses

First, we examined the range and distribution of the total number of valid sentences (see Table 1). As shown in Table 1, the total number of valid sentences differed among the participants; thus, we calculated the proportion of the number of vivid segments and the number of coherent sentences to the total number of valid sentences for each memory and used these ratios as the indicator of vividness and coherence in the subsequent data analyses.

**Table 1:** Range and distribution of number of valid sentences in memories.

	Negative	Positive
	memory	memory
Range of valid sentences	6–283	19–201
N of valid sentences < 25	8.9%	7.7%
25 = < N of valid sentences < 50	22.2%	28.6%
50 = < N of valid sentences < 75	30.0%	36.2%
75 = < N of valid sentences < 100	17.8%	9.9%
100 = < N of valid sentences < 125	11.1%	8.8%
125 = < N of valid sentences	10.0%	8.8%


The descriptive statistics and intercorrelations for the narrative quality (i.e., the ratios of coherent sentences and vivid segments) and attachment dimensions are presented in Table 2. The coherence and vividness scores were positively correlated between negative and positive memories. Specifically, greater coherence for positive memories was associated with greater coherence for negative memories, and greater vividness for positive memories was associated with greater vividness for negative memories. This suggests that participants recalled AM in a similar way across positive and negative emotional contexts. In addition, there was a significant negative correlation between attachment avoidance and the ratio of coherent sentences for negative autobiographical memories. Specifically, greater avoidance was associated with less coherence of negative autobiographical memories.

**Table 2:** Correlations among study variables.

	*M* (*SD*)	2	3	4	5	6
**Attachment dimensions**						
1 Anxiety	2.74 (0.95)	0.44^∗∗^	0.08	–0.11	–0.20	–0.17
2 Avoidance	3.42 (1.32)		–0.18	–0.25^∗^	0.01	0.14
**Narrative quality**						
3 Coherence (positive)	0.20 (0.09)			0.50^∗∗^	0.11	–0.25^∗^
4 Coherence (negative)	0.19 (0.09)				0.08	–0.16
5 Vividness (positive)	0.67 (0.31)					0.35^∗∗^
6 Vividness (negative)	0.59 (0.33)					


*^∗^p < 0.05. ^∗∗^p < 0.01.*

### Relationship Between Attachment Orientation and Narrative Quality

We next conducted a series of hierarchical multiple regression analyses with SPSS 22.0 to test our hypotheses. The ratios of coherent sentences and vivid segments for positive and negative memories were used as dependent variables. In the first step of the regression, verbal ability and rehearsal frequency, which may influence the discourse of memory, were entered as control variables. Grand-mean centered attachment anxiety and avoidance were entered on the second step to test the hypothesized effects of attachment orientation on the quality of AM. The results are presented for negative and positive memories separately.

#### Negative Memory

The results, shown in Table 3, revealed that attachment avoidance was positively associated with vividness of negative autobiographical memories, indicating that adults higher in avoidance had more vivid details in their narratives of negative memories. In addition, attachment avoidance was negatively associated with coherence of negative memories, such that highly avoidant adults had more inconsistent content, incoherent plot, or disfluent speech when narrating their negative memories about events that happened in their married life.

**Table 3:** Regression analyses predicting quality of autobiographical memory by attachment orientation.

	Vividness			Coherence		
Predictors	*β*	*SE*	*p*	*β*	*SE*	*p*
**Negative memory**						
**Step 1**						
Verbal ability	–0.02	0.01	0.099	0.23	0.00	0.030
Rehearsal	–0.18	0.02	0.849	–0.02	0.01	0.870
**Step 2**						
Anxiety	–0.26	0.04	0.025	–0.03	0.01	0.790
Avoidance	0.28	0.04	0.015	–0.24	0.01	0.037
**Positive memory**						
**Step 1**						
Verbal ability	–0.03	0.01	0.816	0.12	0.00	0.260
Rehearsal	0.10	0.03	0.330	0.08	0.01	0.446
**Step 2**						
Anxiety	–0.24	0.04	0.053	0.23	0.01	0.055
Avoidance	0.04	0.04	0.358	–0.30	0.01	0.013


In contrast, attachment anxiety was negatively associated with the vividness of negative memories, indicating that adults higher in attachment anxiety presented less details about their negative memories. However, attachment anxiety was not significantly related to coherence of negative memories.

#### Positive Memory

As shown in Table 3, results revealed that higher attachment avoidance was associated with lower coherence of positive relationship-related memories, indicating that adults higher in avoidance were less likely to narrate their positive memories in a logical and fluent way. Additionally, attachment anxiety was marginally significantly associated with lower vividness and higher coherence of positive memories, indicating that anxious adults narrated their positive relationship-related memories with less details but in a more coherent manner^1^.

## Discussion

The current results revealed significant associations between attachment orientations and the coherence and vividness of AM about married life. Attachment avoidance, but not attachment anxiety, was significantly associated with lower coherence of both positive and negative attachment-related memories and higher vividness of negative memories. Attachment anxiety was associated with lower vividness of negative autobiographical memories, and there was a marginally significant trend for anxiety to be associated with lower vividness and higher coherence of positive memories. These findings suggest that working models of attachment (reflected in attachment orientation) shape the processing and narration of autobiographical memories, especially among avoidant adults. We discuss the coherence and vividness results for each attachment orientation below.

First, as predicted, results indicated that adults higher in avoidance were more likely to present their positive and negative attachment-related autobiographical memories about married life in incoherent narratives. According to attachment theory (McCabe et al., 2006), such incoherent personal narratives may reflect avoidant individuals’ negative IWMs of attachment. That is, the negative schema that avoidant individuals hold of interactions with attachment figures may impede their ability to construct attachment-related memories in a coherent way (Hesse, 2008). Additionally, avoidant adults’ defensive processes, including denial or distortion of thoughts and feelings regarding attachment experiences (Sher-Censor and Yates, 2015), may interfere with their narration of attachment-related experiences and result in narrative descriptions that are off topic or incorrect in logic sequence.

Inconsistent with our prediction, individuals high in avoidance narrated their negative AM with more vivid details. Although this emphasis on negative autobiographical details seems inconsistent with their attempt to deactivate the attachment system, it may reflect their negatively-biased schema-driven processing of attachment-related information. Avoidant adults who hold negative IWMs of others as being unavailable and unresponsive are likely to process attachment-related information in a negative fashion (Dykas and Cassidy, 2011) and may be more likely to remember schema-consistent information. This is consistent with prior research showing that avoidant adults tend to overestimate their partner’s negative emotion during interactions (Overall et al., 2015) and retrieve more negative attachment-related memories (Haggerty et al., 2010; Wang et al., 2017).

These findings suggest that avoidant adults may process attachment-related information in a complex manner, as opposed to simply deactivating memories (which is often concluded when using self-report measures of memory quality) (Niedenthal et al., 2002; Haggerty et al., 2010). Specifically, their defensive attempt to deactivate the attachment system may contribute to their incoherent description (e.g., Sutin and Gillath, 2009), as they may be selectively retrieving memories that were initially biased by their negative attachment schema (e.g., Haggerty et al., 2010). Consequently, avoidant individuals’ memories, especially negative memories, seem to reflect a stacking of fragmented details.

Partly consistent with our prediction, the narrative memories of anxious individuals were less vivid (albeit marginally significant for positive memories), however, there was a marginal trend for anxious individuals to have more coherent positive memories. The high arousal of emotional relationship-related memories (especially negative memories) among anxious individuals may contribute to reductions in the vividness of their narratives (Haggerty et al., 2010; Dykas et al., 2014). It has been reported that intense negative emotion tends to narrow an individual’s attention scope (Huntsinger, 2013) and decreases the amount of extraneous environmental information stored in memory (Biss and Hasher, 2011; Kuhbandner et al., 2011). Accordingly, deficits in recalling details of relationship-related events, as shown by highly anxious adults, might result from an increase in the arousal of emotion.

Additionally, the less strong and inconsistent pattern of results on coherence of positive memories for anxious individuals may reflect the inconsistent working models of attachment that anxiously attached individuals hold. Anxious individuals have both positive and negative aspects to their IWMs (that attachment figures are sometimes responsive and sometimes not) (Mikulincer and Shaver, 2016). Thus, the ambivalence of anxious adults may obscure effects of attachment anxiety on AM. The ambiguous and not robust findings for attachment anxiety suggest that replications and extensions are needed in future research examining attachment influences on autobiographical memories. Future research is also necessary to test potential underlying mechanisms such as emotional arousal.

### Implications and Limitations

A major strength of this investigation resides in the focus on narrative quality of autobiographical memories. Autobiographical narratives reveal the objective content of AM without self-regulation, and thus provide a rigorous test of attachment effects on AM beyond the subjective feelings associated with remembering shown by previous studies. The current results, especially for attachment avoidance, revealed that individuals process narrative memories in ways consistent with their working models of attachment.

A limitation of this investigation is the measure of attachment used in this investigation (see the specific items listed in Appendix 1), which was developed for assessing Chinese older adult’s attachment orientations within the marital relationship. Although conceptually similar, this measure included items for the anxiety dimension that are different from the widely used Experiences in Close Relationships Scale (ECR, Brennan et al., 1998). Thus, the results for attachment anxiety may not generalize to other samples or compare with previous findings directly. Additional research examining the validity of the OAMAS among other samples (e.g., western older adults) and its relationship with the ECR would reveal whether the current results for anxiety are culture-related or age-related, or if they reflect the core nature of attachment anxiety. Furthermore, it will be important for future research to better understand how attachment anxiety, in particular, shapes AM, to gain greater insight into the role of working models of attachment in information processing.

Additionally, a strength of this investigation is its focus on attachment-related memories in the long-term marital relationship of older adults (especially given that older adults have been neglected in this type of research). However, the AM of older adults may differ in important ways from that of younger cohorts, whose AM tends to be less specific, but more coherent overall (Levine et al., 2002; Piolino et al., 2010). Although theoretically we expect attachment orientations to influence the processing of social information in similar ways across the lifespan, given possible age-related differences in the episodic quality of AM, the results of the current study may not be extrapolated to younger adults. Future research is needed to examine the role of attachment orientations in shaping the quality of AM in younger adults to achieve a more comprehensive understanding of this relationship “from the cradle to the grave” (Bowlby, 1973).

## Conclusion

In summary, the results of the current investigation suggest that attachment orientation, especially avoidance, functions schematically in narrating AM of married life in adulthood. This work provides a foundation for future work focused on predictors of the quality of autobiographical memories across the lifespan.

## Ethics Statement

This study was carried out in accordance with the recommendations of the ethical standers of the Institutional Review Board of Faculty of Psychology, Beijing Normal University, Beijing, China. The protocol was approved by the Institutional Review Board of Faculty of Psychology, Beijing Normal University. All subjects gave written informed consent in accordance with the Declaration of Helsinki.

## Author Contributions

YW, QW, and DW wrote the proposal, designed the study, and completed the final draft. YW and QW were involved in data collecting and coder training. YW and BF performed the statistical analysis. BF and DW commented on the second draft. All authors commented on the final manuscript, which was completed by YW, QW, DW, and BF.

## Conflict of Interest Statement

The authors declare that the research was conducted in the absence of any commercial or financial relationships that could be construed as a potential conflict of interest.

## Footnotes

1 As supplementary analyses, hierarchical multiple regression analyses were conducted with the raw number of vivid and coherent sentences as dependent variables. We controlled for the total number of valid sentences because this variable was positively associated with the number of vivid segments (negative: *b* = 0.66, SE = 0.03, *t* = 8.07, *p* < 0.001; positive: *b* = 0.56, *SE* = 0.04, *t* = 6.25, *p* < 0.001) and the number of coherent sentences (negative: *b* = 0.98, *SE* = 0.02, *t* = 55.82, *p* < 0.001; positive: *b* = 0.98, *SE* = 0.02, *t* = 48.77, *p* < 0.001) in the narratives. Results indicated that attachment avoidance was negatively associated with the number of coherent sentences in the positive memory (*b* = –0.05, *SE* = 0.53, *t* = –2.04, *p* = 0.044), marginally positively associated with the vividness of negative narratives (*b* = 0.15, *SE* = 1.22, *t* = 1.71, *p* = 0.090), and marginally negatively associated with the coherence of negative memories (*b* = –0.04, *SE* = 0.54, *t* = –1.83, *p* = 0.070). In contrast, attachment anxiety was marginally negatively associated with the number of vivid segments (*b* = –0.17, *SE* = 1.73, *t* = –1.84, *p* = 0.069).

## Appendix Appendix 1 | Older Adults’ Marital Attachment Scale

Avoidance items:

(1) I don’t want to stay too close to my wife/husband.
(2) I prefer to distance myself from my wife/husband.
(3) I prefer not to be in close contact with my wife/husband.
(4) I get uncomfortable when my wife/husband wants to be very close.
(5) I avoid getting close to my wife/husband.
(6) I prefer to be alone rather than stay with my wife/husband.

Anxiety items:

(1) I worry that my wife/husband will dislike me.
(2) I worry that my wife/husband won’t care about me as much as I care about her/him.
(3) When I show my feelings for my wife/husband, I’m afraid she/he will not feel the same about me.
(4) My wife/husband makes me doubt myself.
(5) My wife/husband only seems to notice me when I’m angry.
(6) I am not sure that I can get support I need from my wife/husband.
